# Adulteration of Food and Medicine

**Published:** 1888-04

**Authors:** 


### ADULTERATION OF FOOD AND MEDICINE.

The oleomargarine legislation of two years ago, and the recent discussions before a Congressional committee, have given the public an idea of the extent to which several important food-products have been adulterated, or mixed with similar substances of inferior quality. The numerous newspaper comments, on these and other phases of the adulteration question, are preparing the people of this country for legislative enactments, such as now protect the people of Great Britain in the purchase of food and drugs.

The English law does not prohibit the sale of mixed or compounded articles, except when unwholesome, but provides that such articles must bear a label, giving their composition in terms which can be understood by the purchaser.

A Chicago spice-grinder sells "selected," "pure," and "strictly pure" pepper, cinnamon, and cloves. The first, or "selected," grade may be adulterated to the extent of 50 per centum, the second to the extent of 10 or 15 per centum, while the third is what the labels indicate. An uninitiated purchaser would expect to find in a "selected" article something even better than "strictly pure." "Refined lard," "creamery butter," and "extract of coffee" are familiar illustrations of a misleading and dishonest use of terms.

Local laws are generally inadequate to correct these evils, but a broad enactment of the National Legislature, compelling manufacturers and dealers to sell food-products for what they are, would give the consuming public general satisfaction.

In several States, and in a few large cities, efforts have been made in the last few years to check the sale of skimmed or watered milk for the pure article. It may be safely said that, in the larger cities and towns, where milk is not regularly examined, 75 per centum is skimmed or watered. What can be done by good laws, well enforced, is shown in the city of Boston.

In 1887, of the samples collected, over 60 per centum were adulterated; in 1884 over 40 per centum were bad; in 1885 30.64 per centum were bad; in 1886 18.55 per centum were bad; while in 1887 the number was reduced to 12.54 per centum; and this with a high standard.

In Chicago something has been done in this direction, and while the results are not as apparent as in Boston, they show, however, that the work begun by Health Commissioner DeWolf is beginning to be felt among the dealers.

The character of the milk sold in this city now is much better than was the case a few years ago.

